# Electronic and optical properties of the buckled and puckered phases of phosphorene and arsenene

**DOI:** 10.1038/s41598-022-24425-w

**Published:** 2022-12-05

**Authors:** Jose Mario Galicia Hernandez, H. N. Fernandez-Escamilla, J. Guerrero Sanchez, Noboru Takeuchi

**Affiliations:** 1 Centro de Nanociencias y Nanotecnología, Universidad Nacional Autónoma de México, Apartado Postal 14, 22800 Ensenada Baja California, México; 2 CICFIM Facultad de Ciencias Físico Matemáticas, Universidad Autónoma de Nuevo León, 66450 San Nicolás de los Garza, Nuevo León México

**Keywords:** Materials science, Optics and photonics, Physics

## Abstract

Using first-principles calculations, we have investigated the structural, electronic, and optical properties of phosphorene and arsenene, group V two-dimensional materials. They have attracted the scientific community’s interest due to their possible applications in electronics and optoelectronics. Since phosphorene and arsenene are not planar monolayers, two types of structures were considered for each system: puckered and buckled arrangements. Computations of band gap were performed within the GW approach to overcome the underestimation given by standard DFT and predict trustable band gap values in good agreement with experimental measurements. Our calculated electronic band gaps lie in the range from near-infrared to visible light, suggesting potential applications in optoelectronics devices. The computed electronic band gaps are 2.95 eV and 1.83 eV for blue and black phosphorene systems. On the other hand, the values for buckled and puckered arsenene are 2.56 eV and 1.51 eV, respectively. Moreover, the study of the optical properties has been dealt by computing the dielectric function imaginary part, which was obtained using the Bethe–Salpeter approach. The use of this technique allows the consideration of excitonic effects. Results indicate strong exciton binding energies of 830 meV for blue phosphorene, 540 meV for black phosphorene, 690 meV for buckled arsenene, and 484 meV for puckered arsenene. The results of our study suggest the possibility of using these materials in electronic and optoelectronic devices.

## Introduction

Graphene is a two-dimensional material with excellent properties, including high carrier mobility, thermal conductance, and mechanical strength, making it an essential material for applications in different industries^1–6^. However, its flat honeycomb structure, with sp^2^ hybridization, makes it less reactive, reducing its possible applications in areas such as catalysis. Unlike graphene, silicene^7^, and germanene^8^ are more reactive due to their atomic buckling. They also lack an intrinsic bandgap, reducing their possible applications in electronic devices, such as field-effect transistors. This is one reason why the search for other 2D materials with new properties is constantly going on. Elements belonging to groups III and V are known to form low-dimensional systems on semiconductor surfaces^9–11^. Therefore, it is not surprising that these elements can also form stable 2D systems. In particular, there is interest in investigating phosphorene and arsenene, a couple of 2D group V materials.

It has been possible to fabricate black phosphorene (puk-P), a monolayer of phosphorus, by exfoliating black phosphorus^12–14^. Its atomic structure is puckered, different from flat graphene, buckled silicene, and germanene. However, another possible phosphorene geometry, similar to that of silicene and germanene, is that of blue phosphorene (buk-P), which was stabilized on an Au (111) substrate^15^. On the other hand, the possible existence of a two-dimensional arsenic system has been investigated^16^. Two structures have been proposed: buckled (buk-As) and puckered (puk-As), similar to blue and black phosphorene, respectively. In all cases, a band gap different from zero has been reported for all monolayers^17–19^.

Puk-P is a 2D layer with high anisotropy due to its different structural arrangements in the armchair and zigzag directions. The anisotropy generates differentiated properties. For example, there exists a clear difference in its effective masses^20^. The strain affects differently the Raman intensities in puk-P if applied either in an armchair or zigzag direction^20^. Also, it shows strong light-matter interactions in the mid-infrared (mid-IR) and visible frequency range because of its excellent carrier mobility, high values of nonlinear saturable absorption coefficients, strong PL emission in the infrared range, anisotropic optical properties, and high-tunable band-gap when stacked one on top of the other^21–23^. These properties allow a wide variety of applications ranging from mid-IR photonics to high-performance field effect devices, such as photodetectors, plasmonic devices with tunable resonance frequency, optical modulators, ultrafast pulse lasers, photodiodes, as well as optical signal processing devices^21,24–27^. Several studies have revealed the stable existence of excitons and trions with high binding energies^23,28,29^, making possible applications in light-emitting and energy harvesting devices^30–32^. In the same way, puk-P is seen as a material with high photoelectrical conversion, making possible the construction of transistors^33,34^. The fabrication of photodetectors with high responsivity in the near-infrared range has been achieved recently^35,36^, as well as phosphorene-based photodetectors integrated into waveguides^35^. Nonperturbative high harmonic generation properties have also been observed in puk-P, suggesting potential applications in extreme ultraviolet and attosecond nanophotonics^27^.

Buk-P possesses almost the same thermal stability as puk-P^37^. Also, its band gap and electron and hole effective masses can be engineered by an electric field^38^. It has been predicted to have high carrier mobilities, a wide absorption spectrum (from infrared to visible), and a strong absorbance in the ultraviolet region^39^, allowing potential applications in electronic and photonic devices, as its band gap lies in the visible range. Its properties can be enhanced when forming vdW heterostructures with GaN, showing a high photocatalytic activity, and allowing applications in optoelectronics and solar energy conversion devices^40,41^. On the other hand, the magnetic and optical properties of buk-P can be significantly improved if it is doped with Y, Nb, Mo, and Zr, since the absorption of infrared and visible light is enhanced in such systems^42^. These results suggest the potential applications of doped buk-P in optoelectronic and spintronic devices. Furthermore, a semiconductor–metal hybrid nanostructure has been reported, consisting of a rectangular 2D array of buk-P inserted into an Au nanowire^39^. In this system, surface plasmon polaritons are observed, allowing the possibility of developing optical modulators, optical waveguides, photosensitive detectors, and nanoscale plasma devices^39^. Besides, high energy conversion efficiencies in the visible region of the electromagnetic spectrum and strong optical absorbance can be reached in other heterostructures based on buk-P, such as buk-P/TMDs, allowing potential applications in thin-film solar cells and light collectors^39,43^.

Ab-initio calculations evidenced that Buk-As, an indirect band-gap semiconductor, is slightly more stable than puk-P and depicts an indirect-to-direct transition when strained up to 6%^16^. It has outstanding optical properties due to its stable buckled structure, making it suitable for optoelectronic applications at room temperature^44^. It possesses a wide band gap, which is useful for fabricating transistors and high-efficiency optoelectronic devices^45^. The band gap can be tuned by atomic and molecular doping as well. By these mechanisms, the optical absorption of buk-As could be possible both in the visible and near-infrared ranges and more important, in the major range of the solar spectrum^45,45^. The enhancement of the optical response makes possible the use of this material in optoelectronic and solar energy harvesting devices^45,46^ for developing technologies based on renewable energy.

Finally, puk-As is an indirect band gap semiconductor. It possesses high carrier mobility and strong in-plane anisotropy as puk-P^47^. As in buk-As, the puk-As presents an indirect-to-direct band gap transition when subject to a strain of just 1%^16^. Because of its outstanding optical properties, potential applications are light-emitting diodes, solar cells, and optoelectronic devices^48–51^. On the other hand, it has been proposed the fabrication of field-effect transistors (FET’s) based on puk-As^52^ of high performance, high on/off ratio, high switching speed, and low energy dissipation. The optical properties of puk-As can also be enhanced by transition metals (TM) adsorption^53^. The TM/arsenene systems can be used to design field emission and photocatalysis nanodevices^53^ because the transition metals can cause an enhancement of the absorption spectrum in the visible and near-infrared regions, which leads to an improvement of the electronic and optical properties for application in visible light catalysis^53^.

As mentioned above, there are several investigations treating the puckered and buckled phases of phosphorene and arsenene separately. However, there is no complete comparative study of their structural, electronic, and optical properties. In this work, we compare their electronic and optical properties using GW and the Bethe–Salpeter approaches, respectively. The study of these properties is very important due to the possible applications of these materials in the electronic and optoelectronic industries.

This paper is organized as follows: Sect. “Computational details” is devoted to a description of the computational details. In Sect. “Results and discussion”, we present and discuss all the results of the structural, electronic, and optical properties. A summary of the results and conclusions is presented in Sect. “Discussion and conclusions”.

## Computational details

The dielectric function imaginary part was computed using the Bethe–Salpeter approach within the scheme of Tamm-Dancoff^54^ to consider excitonic effects. As computational resources concerning G_0_W_0_ and BSE calculations scale exponentially with k-points, we have used a less dense mesh for these computations. However, a thorough convergence study was done, guaranteeing the reliability of our results. Therefore, we used a 12 × 12 × 1 gamma-centered k-points mesh for blue phosphorene and buckled arsenene, and a 10 × 12 × 1 gamma-centered k-points mesh to deal with black phosphorene and puckered arsenene. A convergence test for using these k-point meshed was performed to have a convergence in the computed values of band-gaps in the range of tenths of electron volts. For the GW computations, we considered 100 empty bands per atom. A careful convergence test for using this number of empty bands was also performed, and it was found to be enough, as the number of dealt atoms in all systems is small with low periodicities (1 × 1), and also the number of valence electrons is small. The GW cutoff energy for the response function was set to 480 eV, which controls how many ${\varvec{G}}$ vectors are included in the response function, and the cutoff energy to represent the dielectric matrix was set to 325 eV. To obtain the dielectric function imaginary part within the BSE approach, we used the Haydock algorithm iterative method, the iterative algorithm stops when the difference between two consecutive evaluations of the optical spectra is less than 0.02 a.u. The broadening of the absorption peaks for optical spectra was set to 0.06 eV. The standard density functional theory calculations were performed in the Vienna Ab-initio Package^55–58^ code. The one-electron wavefunctions were treated with the projector augmented wave method as derived by Kresse and Joubert^59^. The electronic states were expanded in a plane wave basis set with an optimized cutoff energy of 680 eV. Exchange–correlation interactions were calculated with the generalized gradient approximation using the Perdew-Burke-Ernzerhof functional form^60^. The following convergence criteria were used: the energy must be lower than 1 × 10^−4^ eV for two consecutive electronic steps, and the norm of all atomic forces must be smaller than 0.007 eV/Å. To evaluate the electronic states at the reciprocal lattice, the Brillouin zone was sampled using an equally distributed^61^ k-points mesh of 17 × 17 × 1.

For finding the quasiparticle energies (quasiparticle energies are referred to the corrected energies of electron system found in standard DFT calculations) within the GW approach, we need to replace the exchange–correlation potential ${v}_{XC}$, used in standard DFT computations by a new operator called self-energy, $\Sigma (\omega )$; which describes the exchange–correlation effects beyond DFT, this operator is non-local and frequency dependent. In this way, the new equation to be solved is:

1 $$\left[-\frac{1}{2}{\nabla }_{i}^{2}+{v}_{ext.}\left(\overrightarrow{r}\right)+{v}_{H}\left(\overrightarrow{r}\right)\right]{\Psi }_{i}\left(\overrightarrow{r}\right)+\int\Sigma \left(\overrightarrow{r},{\overrightarrow{r}}^{^{\prime}};\omega ,{E}_{i} \right){\Psi }_{i}\left({\overrightarrow{r}}^{^{\prime}}\right)d{\overrightarrow{r}}^{^{\prime}}={E}_{i}{\Psi }_{i}\left(\overrightarrow{r}\right),$$

where ${v}_{ext.}\left(\overrightarrow{r}\right)$ and ${v}_{H}\left(\overrightarrow{r}\right)$ are the external and Hartree potential from DFT, $\Sigma$ is the self-energy operator, ${\Psi }_{i}\left(\overrightarrow{r}\right)$ are the quasiparticle eigenstates, and ${E}_{i}$ are the quasiparticle energies.

To find the self-energy it is necessary to find the Green’s function of the interacting system, these two quantities are linked by the equation:

2 $$\Sigma \left(\upomega \right)={G}_{0}^{-1}\left(\omega \right)-{G}^{-1}\left(\omega \right),$$

where is the Green’s function of non-interacting system and is the Green’s function of the fully interacting system. By using the Green’s function (in frequency space) it is possible to know the excitation energies from the poles of next equation:

3 $$G\left(\overrightarrow{r},{\overrightarrow{r}}^{^{\prime}},\omega \right)={\sum }_{i}\frac{{f}_{i}\left(\overrightarrow{r}\right){f}_{i}^{*}\left({\overrightarrow{r}}^{^{\prime}}\right)}{\omega -{\epsilon }_{i}\pm i\eta },$$

where ${f}_{i}\left(\overrightarrow{r}\right)$ are the solutions of quasiparticle equations and can be approximated by Kohn – Sham orbitals ${(\varphi }_{i}^{DFT})$. $i\eta$ represents a small shift to avoid divergences.

Once knowing the Green’s function and self-energy, we can have an expression for quasiparticle energies from results of standard DFT calculations as follows:

4 $${\epsilon }_{i}^{GW}={\epsilon }_{i}^{DFT}+{Z}_{i}\langle {\varphi }_{i}^{DFT}|\left[{\Sigma }_{xc}\left({\epsilon }_{i}^{DFT}\right)-{V}_{xc}^{DFT}\right]|{\varphi }_{i}^{DFT}\rangle ,$$

where ${\epsilon }_{i}^{GW}$ are the quasiparticle energies, ${\epsilon }_{i}^{DFT}$ are the energies from DFT approach, ${\varphi }_{i}^{DFT}$ are Kohn – Sham orbitals, ${\Sigma }_{xc}$ is the self-energy, ${V}_{xc}^{DFT}$ is the DFT exchange–correlation potential and ${Z}_{i}$ is a renormalization factor given by:

5 $${Z}_{i}={\left(1-\frac{\partial {\Sigma }_{xc}}{\partial {\epsilon }_{i}^{DFT}}\right)}^{-1}.$$

To find the self-energy and Green’s function it is necessary to solve a set of coupled integro- differential equations, whose self-consistent solution provides the self-energy of the system and therefore the Green’s functions. These set of equations are the called Hedin’s equations^62^ given by:

6 $$\left\{\begin{array}{c}\Sigma =iGW\\ G={G}_{0}+{G}_{0}\Sigma G\\ \Gamma =1\\ P=iGG\Gamma \\ W=v+vPW\end{array}\right.,$$

where $\Gamma$ is the vertex function, which describes the interactions between virtual hole and electron excitations, $P$ is the irreducible polarizability, which describes the linear response of the density to changes in the total effective potential, $W$ is the dynamically screened interaction between quasiparticles, and $v$ is the bare Coulomb interaction. The self-consistent cycle starts by setting ${G}_{0}=G$, then, the set of equations are iterated until the self-consistency in all terms is reached.

On the other hand, in order to obtain the optical spectra, we need first to solve the Bethe–Salpeter equation in the Tamm-Dancoff approximation for each excitonic state as follows:

7 $$\left({E}_{c{\varvec{k}}}^{QP}-{E}_{v{\varvec{k}}}^{QP}\right){A}_{vc{\varvec{k}}}^{S}+\sum_{{v}^{^{\prime}}{c}^{^{\prime}}{{\varvec{k}}}^{\boldsymbol{^{\prime}}}}\langle vc{\varvec{k}}\left|{K}_{eh}\right|{v}^{^{\prime}}{c}^{^{\prime}}{{\varvec{k}}}^{\boldsymbol{^{\prime}}}\rangle ={\Omega }^{S}{A}_{vc{\varvec{k}}}^{S},$$

where ${E}_{c{\varvec{k}}}^{QP}$ and ${E}_{v{\varvec{k}}}^{QP}$ are the quasiparticle energies obtained from GW approach for the conduction $(c)$ and valence bands $(v)$, ${K}_{eh}$ is known as the electron–hole kernel, ${A}_{vc{\varvec{k}}}^{S}$ is called as the exitonic wave function, and ${\Omega }^{S}$ refers of an excitonic energy. $S$ is referred to an excitonic state.

Once the Bethe–Salpeter equation has been solved, it is possible to compute the dielectric function imaginary part from the excitonic energies found in Eq. (7) as follows:

8 $${\varepsilon }_{2}\left(\omega \right)=\frac{16{\pi }^{2}{e}^{2}}{{\omega }^{2}}\sum_{S}{|\mathbf{e}\cdot \langle 0\left|\mathbf{v}\right|S\rangle |}^{2} ,$$

where $|\mathbf{e}\cdot \langle 0\left|\mathbf{v}\right|S\rangle$ is known as the velocity matrix element, and it is linked with the polarization vector $\mathbf{e}$.

## Results and discussion

This section presents the results of the structural properties of bulk arsenic and phosphorous, followed by the structural optimization of the monolayers derived from the bulk systems. In the next subsections, we focus on the electronic and optical properties of the 2D systems.

### Structural properties

**Figure 1:**
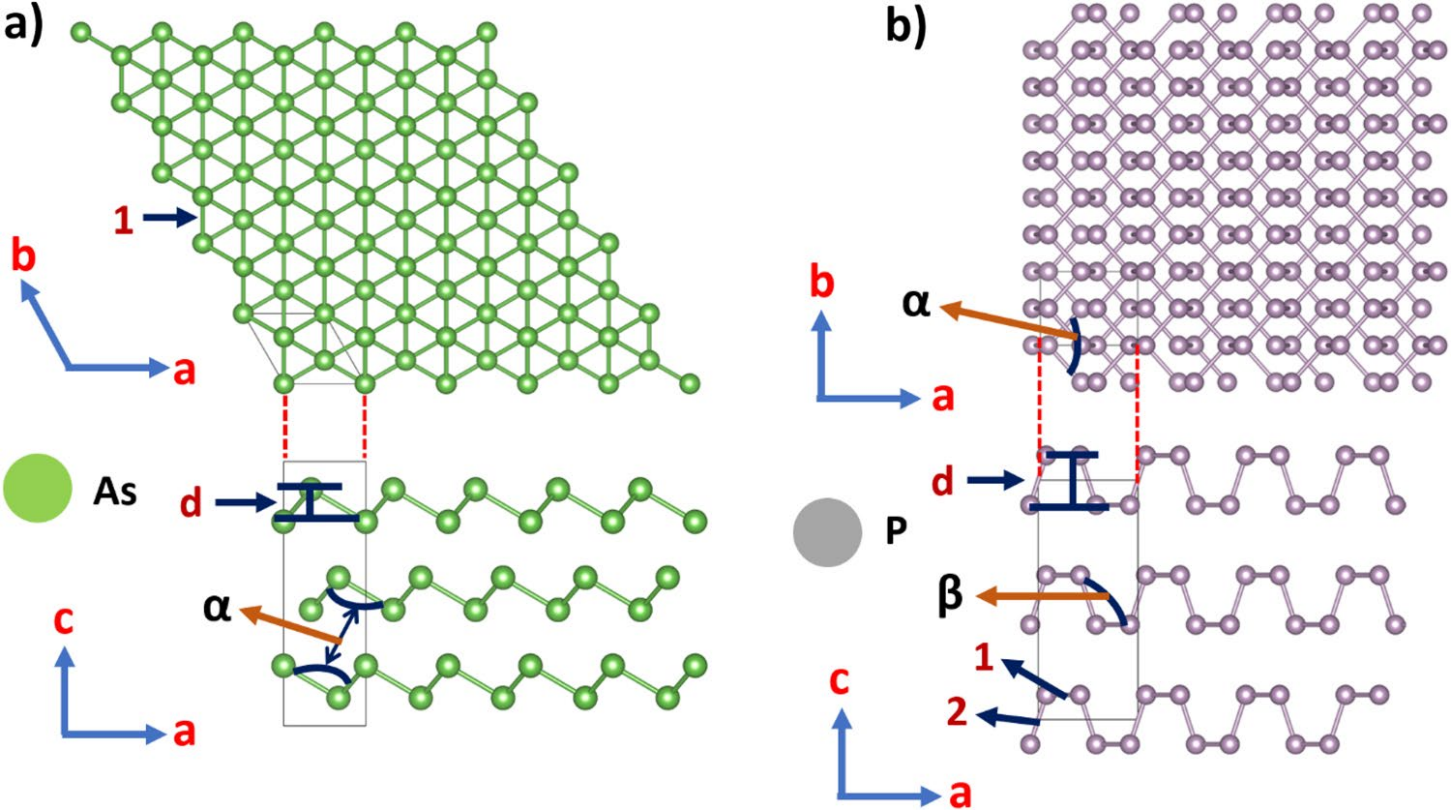
(**a**) Top and side views of bulk arsenic -gray arsenic-, (**b**) and bulk phosphorous -black phosphorus-. 1 and 2 correspond to the bond lengths, α and β correspond to bond angles of Table 1. Figure generated by using the open-source GIMP (GNU Image Manipulation Program) version 2.8.22 (URL: https://www.gimp.org/).

We first optimized the most stable bulk structures for both arsenic and phosphorous. As shown in Fig. 1a, the most common structure for As is the gray phase in which the atoms form a double-layered structure consisting of interlocked, six-membered rings. As summarized in Table 1, our calculated parameters agree with the experimental values from Ref^63^. On the other hand, as shown in Fig. 1b, the most thermodynamically stable structure for bulk P is the black phase with an orthorhombic pleated honeycomb structure, in which each atom is bonded to three other atoms. As summarized in Table 1, our calculated parameters are in good agreement with experimental values from Ref^64^.

**Table 1:** Structural parameters and cohesive energies of puckered and buckled phosphorene and arsenene.

Structure	Cohesive energy (eV/atom)	Lattice constant (Å)		Bond length	Bond angle
		a	B		
Black (Puckered) phosphorene	− 2.954	4.62	3.29	1:2.22	1: 95.93
				2: 2.25	2: 104.15
				d = 2.10	
Blue (Buckled) phosphorene	− 2.917	3.27	3.27	1: 2.26	1: 92.92
				d = 1.23	
Bulk phosphorus	− 3.198	4.37 (^b^4.37)	3.31 (^b^3,31)	1: 2.22	1: 96.37
			c = 10.47	2: 2.27	2: 101.91
			(^b^c = 10.36)		
Puckered arsenene	− 3.481	4.76 (^c^ 4.76)	3.68	1: 2.50	1: 94.68
			^c^3.67	2: 2.49	2: 100.76
				d = 2.39	(^c^1: 94.64
				(^c^1: 2.501	2: 100.80)
				2: 2.485)	
Buckled arsenene	− 3.516	3.60 (^c^ 3.607)	3.60	1: 2.50	1: 92.10
				(^c^1: 2.503)	(^c^92.22)
				d = 1.39	
Bulk arsenic	− 3.345	3.75 (^a^3.7598)	3.75	1: 2.51	1: 96.66
			c = 10.44		
			(^a^c = 10.5475)		

Parameters of black phosphorous and gray arsenic are also presented. Bulk experimental values are taken from (a) Ref.^63^ and (b) Ref.^64^. Previous arsenene values are taken from Ref.^16^.

As mentioned in the introduction, phosphorene has been stabilized in two different phases, blue (buckled) and black (puckered) phosphorene. Blue phosphorene and buckled arsenene belong to space group No. 164 $\left(P\overline{3 }m1\right)$. On the other hand, black phosphorene and puckered arsenene belong to space group No. 53 $\left(Pmna\right)$.

**Figure 2:**
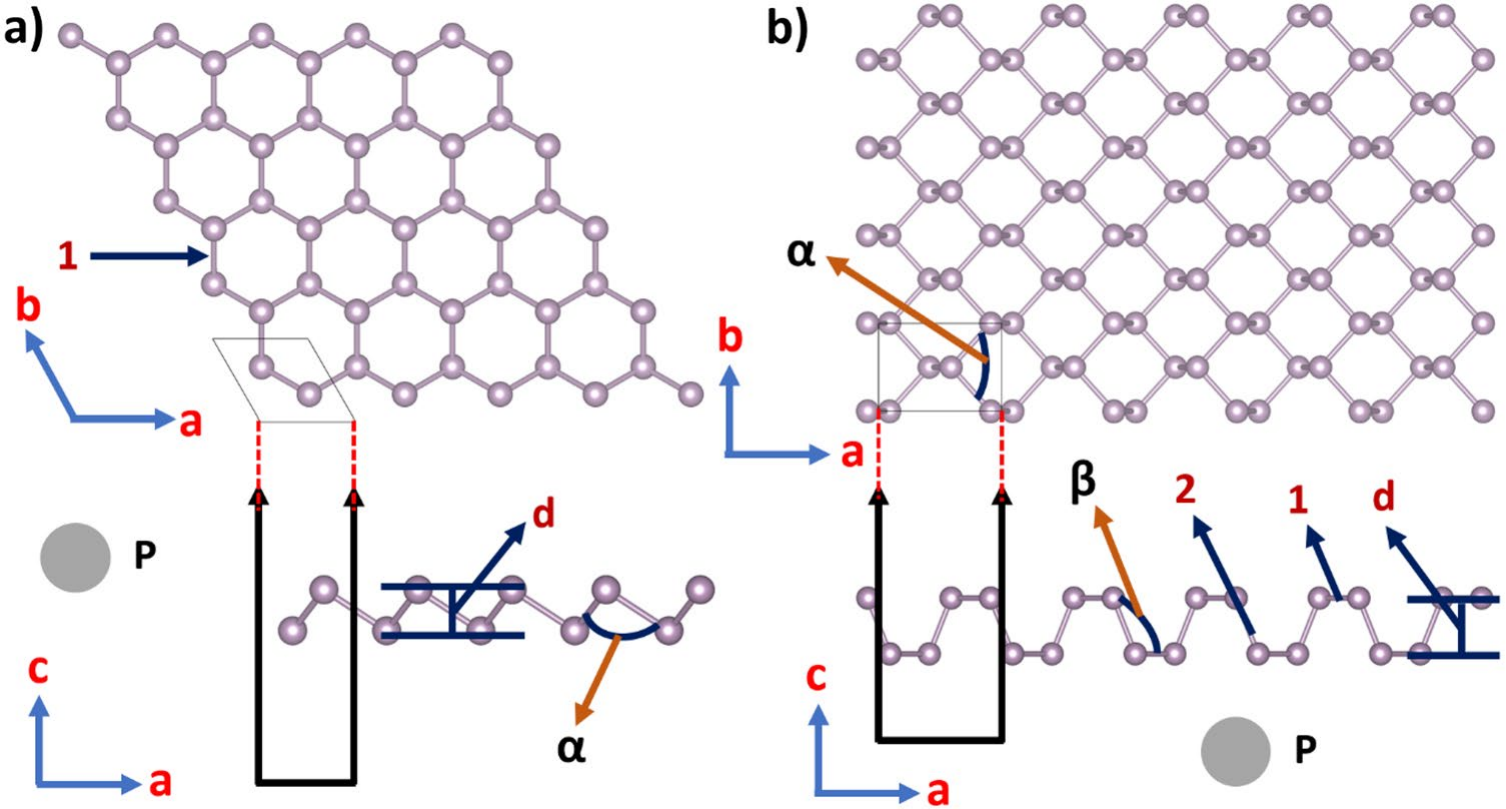
(**a**) Top and side views of blue (buckled) phosphorene, (**b**) Top and side views of black (puckered) phosphorene. 1 and 2 correspond to the bond lengths, α and β correspond to bond angles of Table 1. Figure generated by using the open-source GIMP (GNU Image Manipulation Program) version 2.8.22 (URL: https://www.gimp.org/).

**Figure 3:**
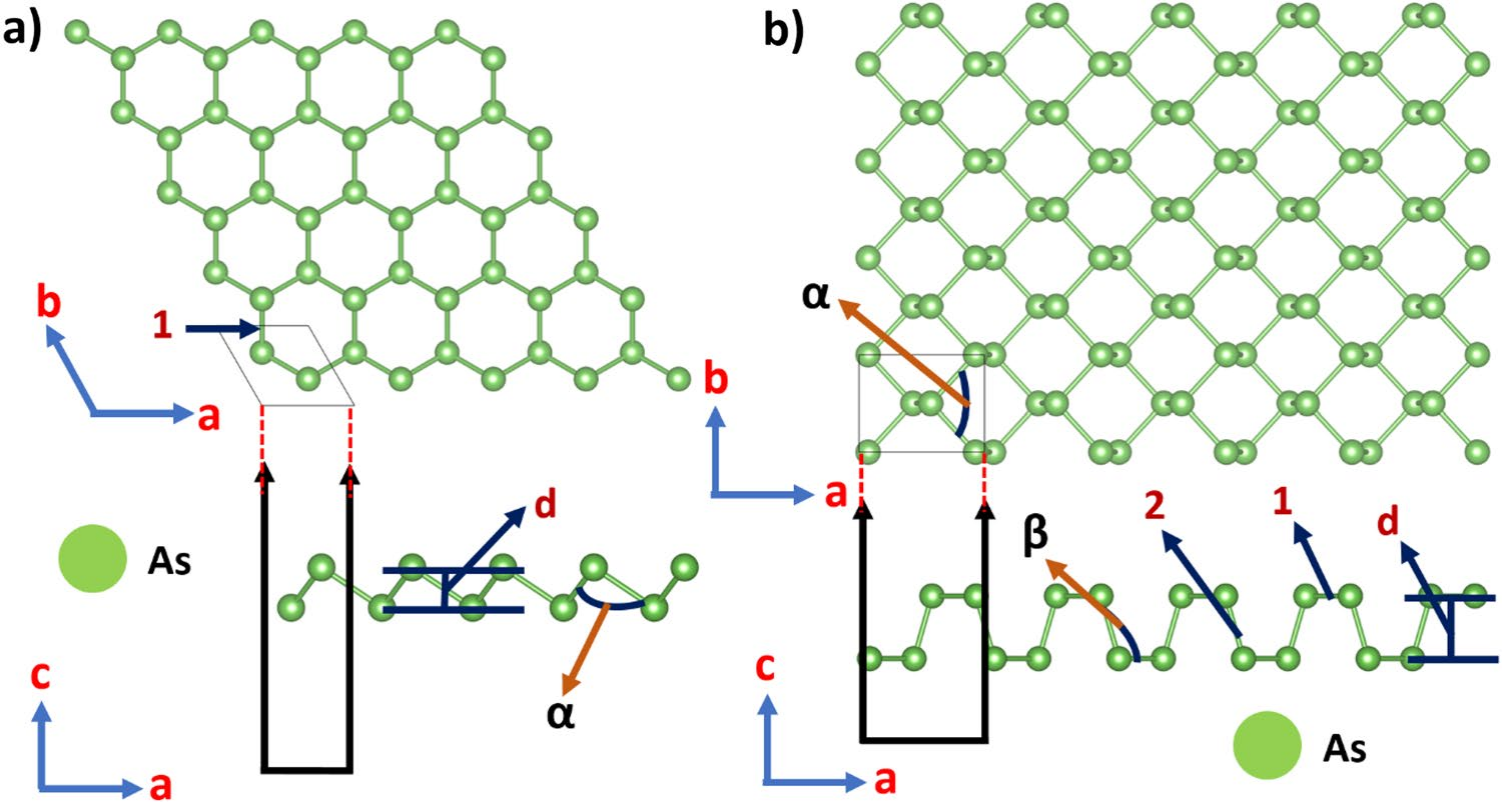
(**a**) Top and side views of buckled arsenene, (**b**) Top and side views of puckered arsenene. 1 and 2 correspond to the bond lengths, α and β correspond to bond angles of Table 1. Figure generated by using the open-source GIMP (GNU Image Manipulation Program) version 2.8.22 (URL: https://www.gimp.org/).

A summary of the structural parameters is presented in Table 1. For puk-P (Fig. 2b), the lattice constants are slightly changed when going from 3 to 2D: a lattice constant is elongated and b is slightly shortened, but the bond lengths are practically unaffected. Finally, the bond angles and buckling distance (d) are slightly more affected because of the breaking of the periodicity along the direction perpendicular to the *xy* plane in the 2D system. In buk-P (Fig. 2a), the bond lengths are quite similar to the ones of its bulk counterpart. Lattice constants, bond angles, and buckling distance (d) differ more significantly because buk-P belongs to another space group (hexagonal). On the other hand, bulk arsenic belongs to a hexagonal space group. For this reason, the structural properties of buk-As (see Fig. 3a) such as lattice constants and bond lengths are similar to their bulk counterpart because both systems have hexagonal symmetry. Bond angles and buckling distance (d) differ from bulk because of symmetry breaking along the c-axis in the 2D system. Finally, the structural properties of puk-As (see Fig. 3b) differ significantly from bulk arsenic since the 2D system belongs to another space group (tetragonal). Our results are in very good agreement with previous calculations found in the literature. Other structural parameters are summarized in Table 1. This table also shows the cohesive energies of the studied structures. Their values reveal that pukered and buckled versions of each 2D system have very similar cohesive energies, and these values are close to those of their corresponding bulk counterparts, suggesting that all 2D systems could be obtained experimentally.

### Electronic properties

**Figure 4:**
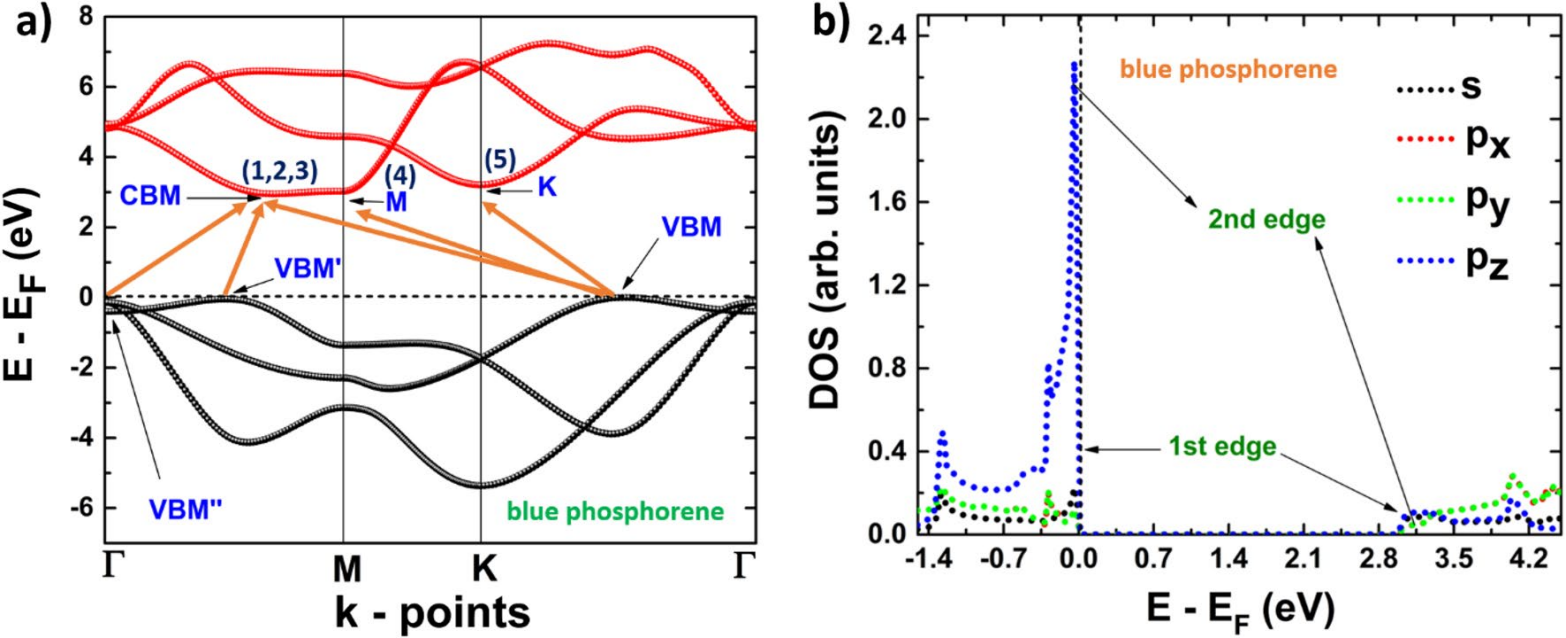
(**a**) Band structure;1, 2, 3, 4 and 5 refers to possible electronic transitions. (**b**) projected density of states of blue phosphorene.

To study and describe the electronic properties of the 2D systems, we have computed the electronic band structures and the projected densities of states. Firstly, buk-P is a semiconductor with an indirect band gap. Its valence band maximum (VBM) is located at an intermediate point between K and Γ, while its conduction band minimum (CBM) is at an intermediate point between Γ and M (see Fig. 4a). However, there are several local maxima in the valence band with similar energies and several local minima in the conduction band with very close energy values, making possible the occurrence of various indirect transitions. The last valence band shows three different band edges, the first one is located at VBM, the second and third are located at Γ (VBM’’), and in an intermediate point (VBM’) between Γ and M, respectively, corresponding to two local minima. Figure 4b is helpful to determine how the three valence bands are formed. The contribution to form these edges can be explained as follows: the first edge is located precisely at the Fermi level, and it is formed by *s*, *p*_*z*_, *p*_*x*_ and *p*_*y*_-orbitals. The contributions of the second and third edges can be seen from the sharp peak formed by *p*_*z*_-orbitals located very close to the Fermi level. As seen in Fig. 4b, the energy difference between the Fermi level and the energy at which the sharp peak is located is very small, in agreement with the band structure. On the other hand, the first conduction band shows also three different edges, the first one, located at the CBM, comes from the contribution of *p*_*z*_-orbitals, while the second (located at M), and third (located at K) are formed by *s*, *p*_*x*_ and *p*_*y*_-orbitals. With this information in mind, we can identify the first five electronic transitions from the last valence band to the first conduction band. These transitions are labeled as follows: 1 from VBM to CBM, 2 from VBM’ to CBM, 3 from VBM’’ (Γ) to CBM, 4 from VBM to M, and 5 from VBM to K.

**Figure 5:**
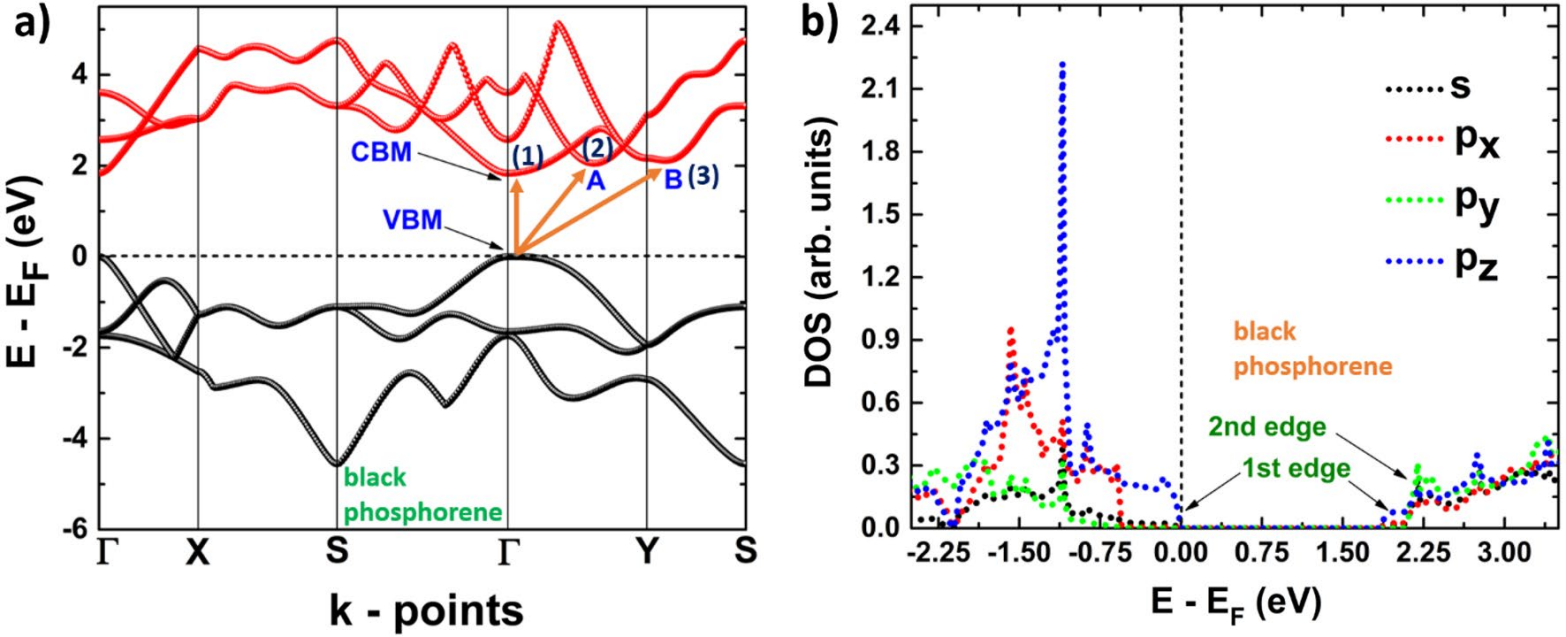
(**a**) Band structure; 1, 2, and 3 refer to possible electronic transitions. (**b**) projected density of states of black phosphorene.

On the other hand, puk-P behaves as a direct band-gap semiconductor (see Fig. 5a), with valence band maximum and conduction band minimum both located at the Γ point, giving place to electronic transition 1. There are two additional local minima in the first conduction band, one located at an intermediate point between Γ and Y, labeled as A, and the other at a point along Y and S, labeled as B. This fact makes possible the existence of two indirect transitions, from Γ to each local minimum (transitions 2 and 3). Figure 5b shows the orbital contributions to the bands, with the edge of the last valence band formed by *p*_*z*_-orbitals. The first conduction band is observed to have three different edges, the first located at Γ and formed by *p*_*z*_-orbitals. The second, located at A, and the third at B, are both formed by *s*, *p*_*x*_ and *p*_*y*_ orbitals.

**Figure 6:**
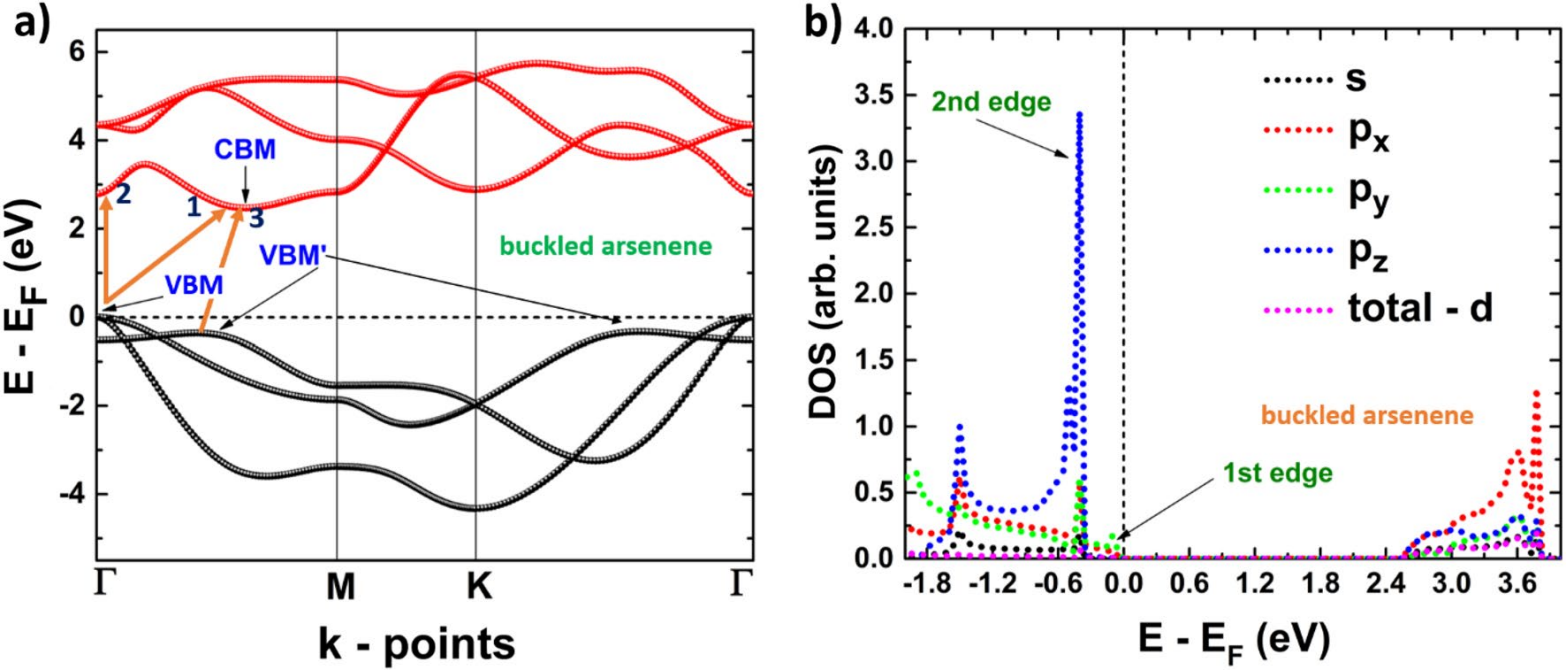
(**a**) Band structure; 1, 2 and 3 refers to possible electronic transitions. (**b**) projected density of states of buckled arsenene.

Let us now focus on the arsenene systems. Buk-As is also an indirect band-gap semiconductor, with the valence band maximum located at the Γ point and the conduction band minimum at an intermediate point between Γ and M (see Fig. 6a), allowing the main indirect transition (1). However, a quasi-direct transition can be possible (2), as a local minimum in the conduction band is observed at the Γ point. The energy difference between these two gaps is small, so both transitions are possible. In the same way, as shown in the band structure, a second indirect transition can take place (3), from an intermediate point between Γ and M, labeled as VBM’ to CBM. Another local maximum in the last valence band is also observed and located in an intermediate point between K and Γ. The energy level of this point is the same as VBM’, and also indirect transitions from this point to CBM are possible. Besides, the last valence band shows three edges, the first located at Γ and formed by *p*_*y*_-orbitals, the other two located at both VBM’ points and formed by *s*, *p*_*x*_, *p*_*y*_ and *p*_*z*_-orbitals, see Fig. 6b.

**Figure 7:**
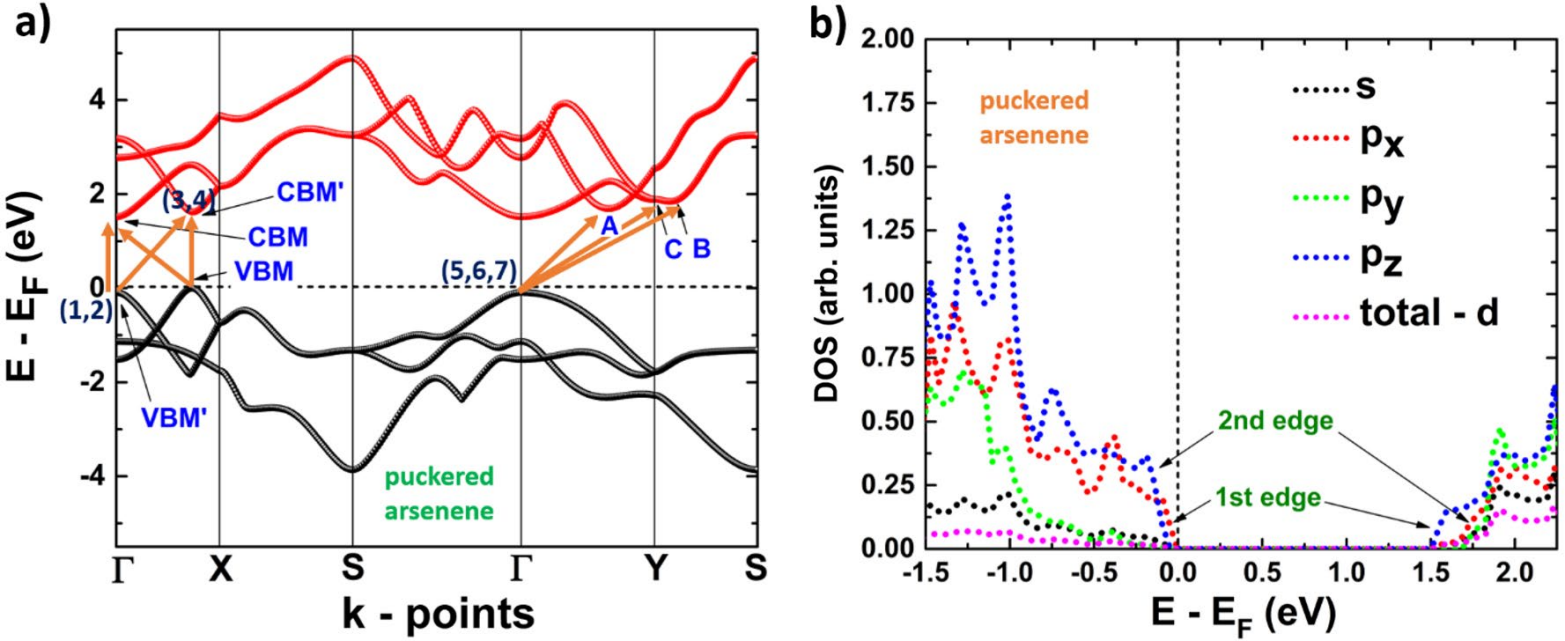
(**a**) Band structure; 1, 2, 3, 4, 5, 6 and 7 refers to possible electronic transitions. (**b**) projected density of states of puckered arsenene.

Finally, in puk-As, the same behavior is observed. From the electronic band structure, we can see that the system is an indirect band gap semiconductor (see Fig. 7a), where the valence band maximum (VBM) is located at an intermediate point between Γ and X, and the conduction band minimum (CBM) is located at the Γ point, resulting in an indirect transition (1). However, two quasi-direct transitions are possible, one at the Γ point (2) and a second one from VBM to a local minimum in the conduction band located at the same point than VBM and labeled as CBM’ (3). Besides, there is also another possible indirect transition from Γ to CBM’ (4). The energies of these transitions are basically the same, so they have the same probability of occurrence. From the band structure, it is possible to identify three points located in the conduction band and very close in energy to CBM’ and Γ. These points are labeled as A, B, and C. From this, it is expected that three indirect transitions (5, 6, and 7) are also likely to occur, from Γ to A, B, and C. Figure 7b helps us to understand how the two edges of the last valence band and first conduction band are formed. The first edge of the valence band located at VBM is formed by p_*x*_-orbitals, while pz-orbitals form the second edge placed at Γ. On the other hand, the first edge of the conduction band located at the points Γ and CVM is formed by *p*_*z*_-orbitals, while the second edge is formed by *s*, *p*_*x*_ and *p*_*y*_-orbitals.

It is worth mentioning that band structures as well as projected densities of states were computed within the GW approach. In this way, it was possible to compute the energies of band-gap and the ones of other possible electronic transitions by considering the GW approximation. A summary of this information can be found in Table 2.

**Table 2:** Energies of electronic transitions of phosphorene and arsenene systems computed within the G_0_W_0_ approach, (i) and (d) denote indirect and direct transitions respectively.

2D system	Transition energy (eV)
Blue phosphorene	1: 2.95 (i) ***band-gap***
	2: 2.98 (i)
	3: 3.02 (i)
	4: 3.09 (i)
	5: 3.21 (i)
Black phosphorene	1: 1.83 (d) ***band-gap***
	2: 2.05 (i)
	3: 2.11 (i)
Arsenene (buckled)	1: 2.56 (i) ***band-gap***
	2: 2.90 (d)
	3: 2.94 (i)
Arsenene (puckered)	1: 1.51 (i) ***band-gap***
	2: 1.55 (d)
	3: 1.62 (d)
	4: 1.71 (i)
	5: 1.78 (i)
	6: 1.93 (i)
	7: 1.97 (i)

From Table 2, we can conclude that our findings agree with previous results for monolayers and heterostructures based on these materials^65–67^.

Significant values are in bold.

### Optical properties

In order to study the optical properties of the 2D systems, we have computed the dielectric function imaginary part within the Bethe–Salpeter approximation, which takes into account excitonic effects. Therefore, using this approach, it is possible to compute the exciton binding energy, by following the expression:

9 $${E}_{be}={E}_{fg}-{E}_{og},$$

where, ${E}_{be}$ refers to the exciton binding energy, ${E}_{fg}$ is the energy of the fundamental gap (direct or indirect as it corresponds to each case), and ${E}_{og}$ is the energy of the optical gap. This last term can be obtained from the dielectric function imaginary part, the energy at which the first peak is observed. In summary, the exciton binding energy is the difference between the fundamental and optical gaps. For energies between the optical and fundamental gaps, the system is excited, and the creation of an exciton takes place, i.e. an electron–hole coupled system exists. If the excitation energy exceeds the fundamental gap, the system decouples, and the excited electron occupies the conduction band.

**Figure 8:**
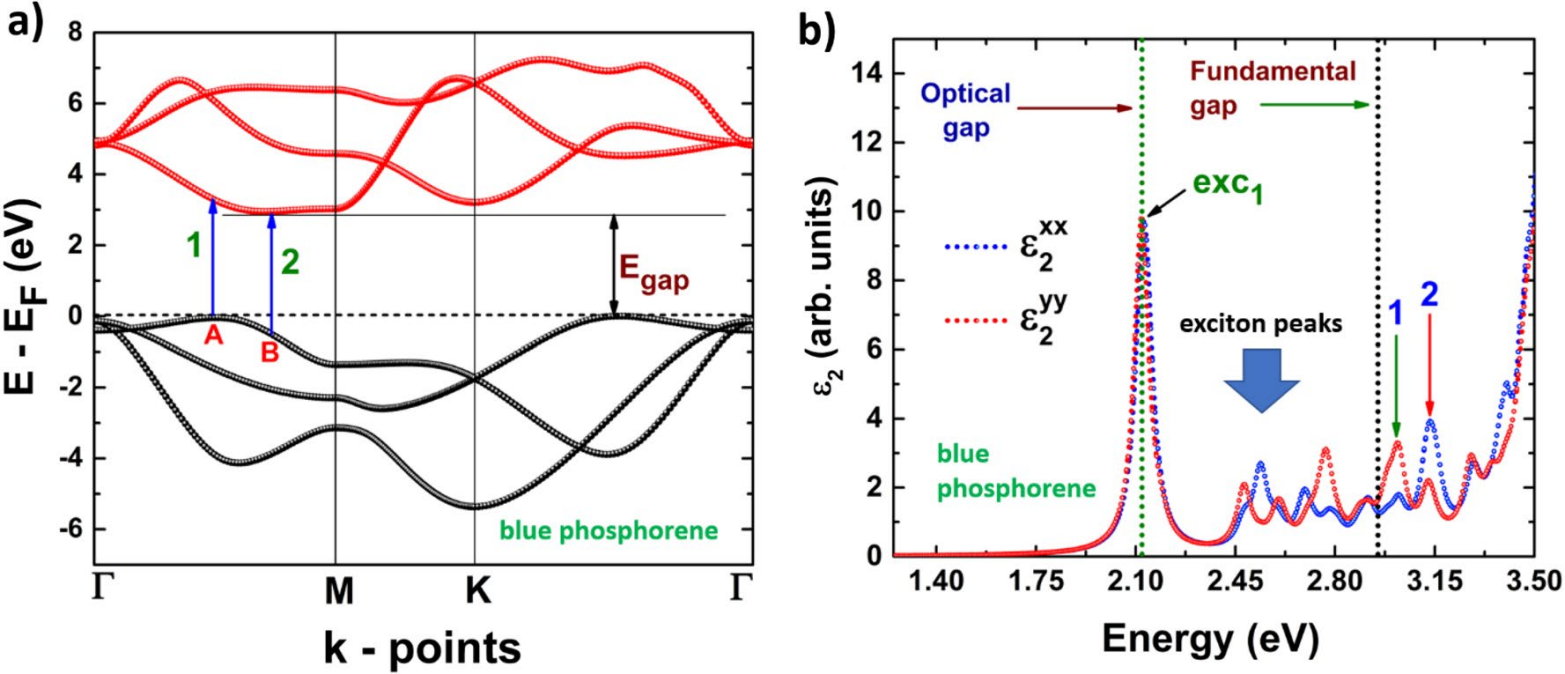
(**a**) Electronic band structure of blue phosphorene including the information of two important direct transitions. (**b**) Dielectric function imaginary part of blue phosphorene showing the values of the corresponding optical and fundamental gaps as well as exciton peaks. The peaks associated with direct transitions depicted in (**a**) are also included.

**Figure 9:**
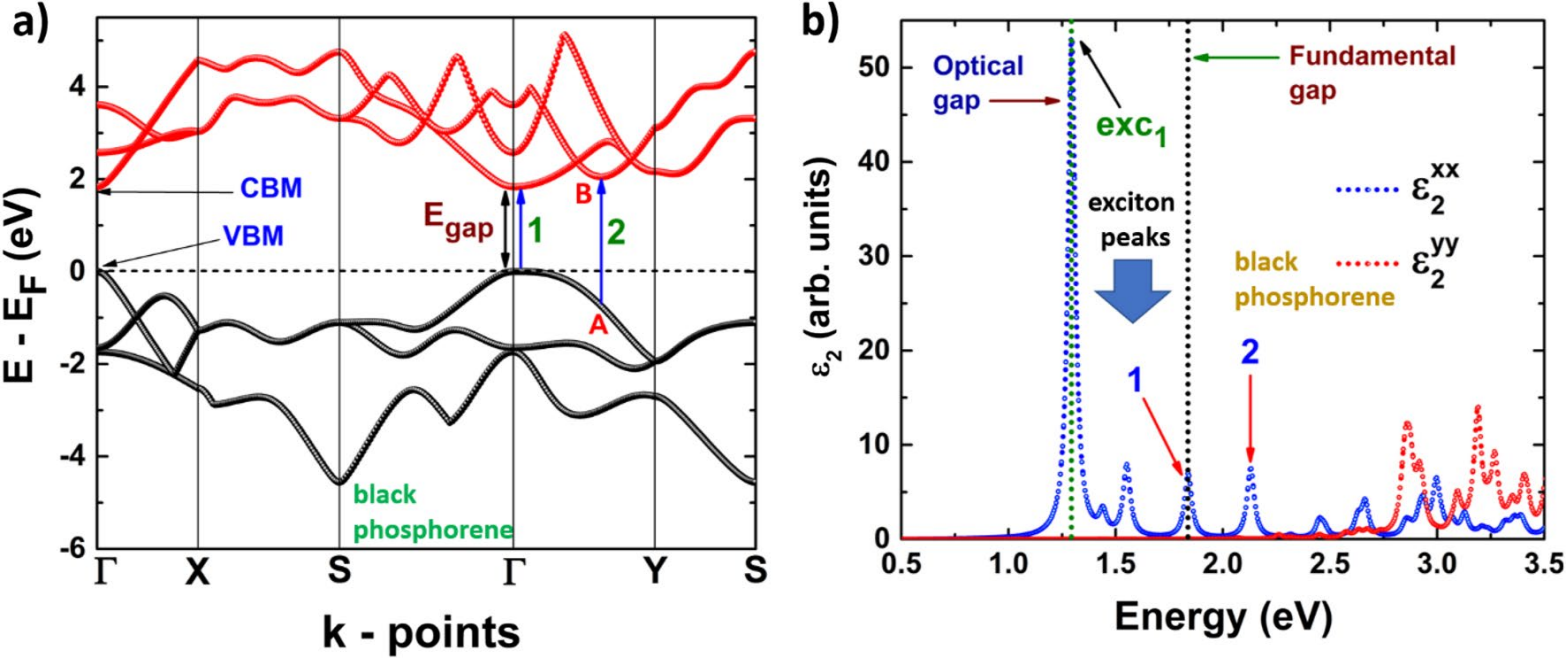
(**a**) Electronic band structure of black phosphorene including the information of two important direct transitions. (**b**) Dielectric function imaginary part of black phosphorene showing the values of the corresponding optical and fundamental gaps as well as exciton peaks. The peaks associated with direct transitions depicted in (**a**) are also included.

**Figure 10:**
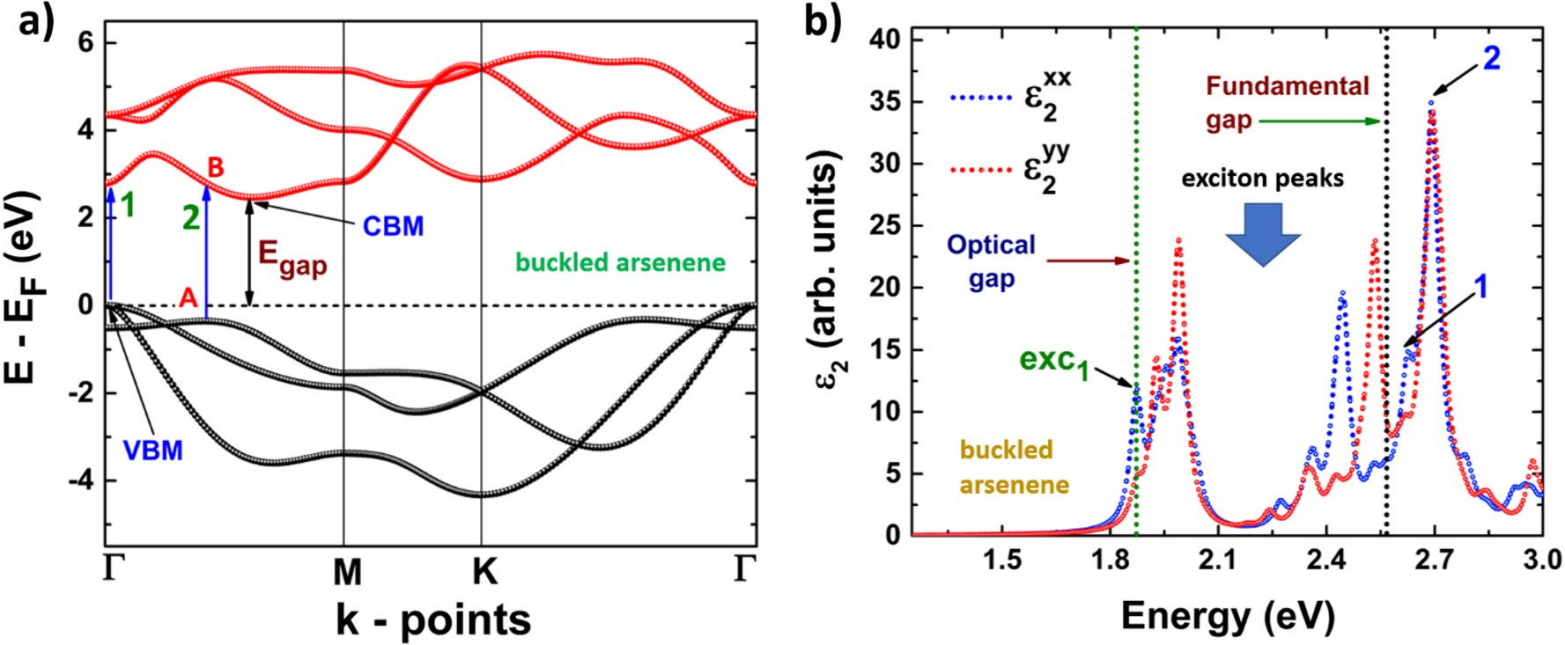
(**a**) Electronic band structure of buckled arsenene including the information of two important direct transitions. (**b**) Dielectric function imaginary part of buckled arsenene showing the values of the corresponding optical and fundamental gaps as well as exciton peaks. The peaks associated with direct transitions depicted in (**a**) are also included.

**Figure 11:**
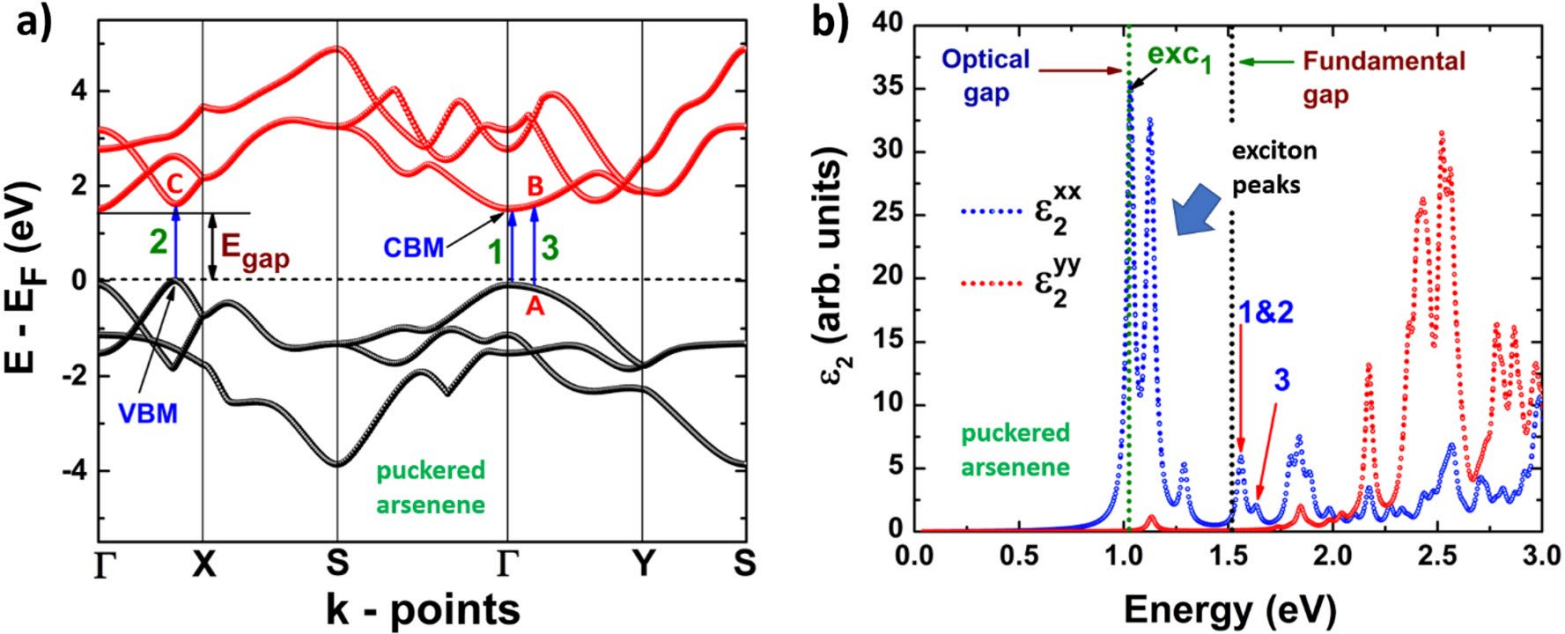
(**a**) Electronic band structure of puckered arsenene including the information of two important direct transitions. (**b**) Dielectric function imaginary part of puckered arsenene showing the values of the corresponding optical and fundamental gaps as well as exciton peaks. The peaks associated with direct transitions depicted in (**a**) are also included.

Figures 8b, 9b depict the dielectric function imaginary part of phosphorene systems, blue and black, and Figs. 10b, 11b are devoted to buckled and puckered arsenene systems. In these figures, we have also included the electronic band structures with relevant information to explain the plots concerning the dielectric function imaginary part. This information is related to the first direct electronic transitions from the last valence band to the first conduction band. The energy associated with these transitions can be seen in the dielectric function imaginary part as the energy position of different peaks above the value of the fundamental gap, i.e. the energy of each peak corresponds to the energy of some direct transition. The BSE approach reveal the information concerning only to the direct transitions, as indirect transitions involve other mechanism not considered in the BSE optical spectra. Therefore, the optical response related to indirect transitions has been neglected in this part of the study. We have considered de *xx* and *yy* components of the dielectric tensor to describe light-mater interactions. In this way, *xx* refers to polarization along x-axis, and yy refers to polarization along y-axis. We have not included the z-direction as we are dealing with 2D systems and the xx and yy components provide the main information about the optical response.

In general, blue phosphorene is considered as an isotropic material, i.e. its physical properties are basically the same when measured along *x* or *y* directions. However, from Fig. 8b it can be seen that the optical response is slightly different, especially at energies higher than the fundamental gap: the positions of the peaks are basically located at the same energies, but the intensities are different. From Fig. 8b, we can see that the optical gap in blue phosphorene is equal to 2.12 eV, which is the energy at which the first peak is observed, and it is equal to the energy of the first exciton level (ground state). This peak is the same for *xx* and *yy* components. We can also observe the presence of ten other exciton levels: five are due to polarization along the *x*-axis, and the other five are due to polarization along the *y*-axis. In Fig. 8b, information about two important direct electronic transitions is also provided. They are labeled as 1 and 2 in Fig. 8a: 1 is the direct transition from a local maximum in the valence band located in an intermediate point between Γ and M (labeled as A), and direct transition 2 is from the point labeled as B to the conduction band minimum.

The optical response of black phosphorene is depicted in Fig. 9b. We can clearly see an anisotropic behavior of the optical response. If the incident excitation is along the *y*-axis, the response is generated for energies around 2.85 eV, as the first peak for this polarization is observed near that energy. However, if the polarization is along the *x*-axis, the optical response is seen for lower energies, the first peak is observed at 1.29 eV, which is equal to the optical gap and it correspond to the first exciton level. The contribution to this peak comes from the polarization along the *x*-axis, as well as from the two following excitonic peaks. In this way, the excitonic peaks are only coming from polarization along *x*-axis, with no contribution from polarization along *y*-axis. As the electronic behavior of the system is direct, the peak located at 1.83 eV is attributed to the direct electronic transition (labeled as 1 in Fig. 9b) at the Γ point, and it corresponds to the value of the fundamental band gap. On the other hand, from Fig. 9a, it can be observed a local minimum in the first conduction band in an intermediate point located between Γ and Y (labeled as B). Therefore, a direct transition to this point is possible, from a point labeled as A in the last valence band. This direct transition is labeled as 2 in Fig. 9a, and it is referred to the peak, labeled also as 2, in the dielectric function imaginary part.

Results of the optical response of buckled arsenene are depicted in Fig. 10b. We can observe that the optical response is not isotropic when the polarization is along the *x* or *y* axis. However, the response is similar to blue phosphorene: the peaks are located at the same energy, but the intensities differ. The first peak, which corresponds to the optical gap, is located at 1.87 eV, and it also corresponds to the first exciton level. This peak is mainly attributed to the polarization along the *x*-axis, although a slight contribution comes from the polarization along the *y*-axis. We can observe several additional excitonic levels: six attributed to polarization along the *x*-axis, and the other six to polarization along *y*-axis. Figure 10a depicts two important direct transitions: the first one, labeled as 1, takes place at the Γ point, and its energy corresponds to the energy position of the peak labeled as 1 in the dielectric function imaginary part. Also, in Fig. 10a we can observe a local valence band maximum in an intermediate point between Γ and M, labeled as A. Therefore, a direct transition from this point to the one labeled as B in the first conduction band can be possible. This transition is referred to as the peak labeled as 2 in the dielectric function imaginary part.

Finally, the results of the optical response for puckered arsenene are depicted in Fig. 11b. We can observe a clear anisotropic behavior. The system slightly responds if the polarization is along the *y*-axis at energies lower than 2.0 eV. Consequently, the optical response for low energies of puckered arsenene is due to polarization along the *x*-axis. The first peak in the dielectric function appears at 1.02 eV (optical gap), corresponding to the first exciton level. Three more exciton peaks due to polarization along the x-axis are observed, and just one at low intensity is attributed to polarization along the y-axis. In Fig. 11a it can be seen two direct transitions with basically the same probability of occurrence since the energies associated with these transitions are very similar. The first of these transitions occur at the Γ point (it is labeled as 1 in Fig. 11a), and it corresponds to the peak in dielectric function imaginary part labeled with the same name. The second direct transition (labeled as 2) takes places from the VBM to a point located in the first conduction band labeled as C. The corresponding peak in the optical spectra attributed to this transition is the same as the one of transition 1, since as mentioned, their transition energies are very similar for both. Finally, we can observe a low dispersion region of the last valence band and the first conduction band, in the Γ–Y path, near the Γ point. It suggests the existence of a direct transition involving two points in this special region. These two points are labeled as A and B in Fig. 11a, and the direct transition between these points is referred as peak 3 in the dielectric function imaginary part.

Finally, the exciton binding energies computed by Eq. (9) are summarized in Table 3. We can observe high value binding energies, with the same order of magnitude found in other 2D monolayers.Number in parenthesis refer to the energy position (in eV) of the first exciton peak (ground state exciton) in the corresponding dielectric function imaginary part. These values correspond to each optical gap.

**Table 3:** Exciton binding energies of phosphorene and arsenene systems.

2D system	Exciton binding energy (meV)
Blue phosphorene	830 (2.12)
Black phosphorene	540 (1.29)
Arsenene (buckled)	690 (1.87)
Arsenene (puckered)	484 (1.02)

## Discussion and conclusions

We have studied the formation of two-dimensional systems for group V elements phosphorene and arsenene. Two stable structures were found for each material: a puckered geometry in which each atom bonds with three other atoms, forming a quadrangular pyramid, and a buckled configuration in which each atom also bonds with three other atoms but in an arrangement similar to silicene and germanene. For both phosphorene and arsenene, the cohesive energies of puckered and buckled configurations are almost the same, indicating the possible existence of both. Due to the larger arsenic atomic radius, lattice constants and bond lengths are larger for arsenene than phosphorene.

Regarding the electronic properties, arsenene and phosphorene are semiconductors in both phases. Blue phosphorene and buckled arsenene have indirect band gaps of 2.95 and 2.56 eV, respectively. Minor differences can be observed in the band structures. The valence band maximum in blue phosphorene is between K and Γ, while in buckled arsenene is at the Γ point. Although the conduction band minimum in blue phosphorene and buckled arsenene is between Γ and M, for buckle arsenene there is another local minimum at Γ, with very similar energy. As a consequence, in buckled arsenene there is a direct transition of 2.9 eV. Other indirect transitions can be seen for both, blue phosphorene and buckled arsenene. In the case of black phosphorene, the band gap is direct at the Γ point with a value of 1.83 eV, while in the case of puckered arsenene, the band gap is indirect with a value of 1.51 eV. For both, black phosphorene and puckered arsenene, the electronic band structures show more than one edge for the last valence band and the first conduction band. This fact allows several direct and indirect transitions with very similar probability of occurrence. The fundamental band gaps of arsenene are smaller than those of phosphorene, indicating a more metallic behavior.

Finally, we have computed the imaginary part of the dielectric function. Results show that the optical response is mostly isotropic in hexagonal systems (blue phosphorene and buckled arsenene), and clearly anisotropic in tetragonal ones (black phosphorene and puckered arsenene). The computation of dielectric function allows us to understand the details concerning the electronic transitions, which are directly related to the dielectric function's peaks. The calculation of dielectric function also allows us to calculate the exciton binding energies. The calculated values for blue phosphorene and buckled arsenene are 830 and 590 eV, while for black phosphorene and puckered arsenene are 540 and 484 eV, respectively. These exciton binding energies are high, as reported for other 2D monolayers. Since exciton binding energies give stability against thermal dissociation, our results show that both phosphorene and arsenene, in buckled and puckered configurations, are materials with suitable properties for optoelectronic devices.

Our results suggest that the electronic properties of all systems are attractive for several applications because several electronic transitions are probable to occur, although the systems with indirect transitions are restricted for some applications; in the systems dealt in this work, we have observed the presence of special aspects in the electronic band structures, suggesting that quasi-direct electronic transitions can occur. This fact makes possible the applications of these systems in several electronic devices. Finally, all systems show a good optical response in the range of visible and near-infrared electromagnetic spectra, which makes possible several applications in photonic and optoelectronic devices.

## Data Availability

The data used to obtain the results of the current study is available from the corresponding author on reasonable request.
